# A systematic review of implementation strategies to improve timely initiation of antenatal care among pregnant women in sub-Saharan Africa

**DOI:** 10.4102/jphia.v17i1.1461

**Published:** 2026-03-26

**Authors:** Olatubosun Akinola, Rabson Zimba, Kutha Banda, Caroline L. Mangani, Hilda Shakwelele, Ibrahim Abdallah, Angel Mwiche, Cindy Chirwa, Mutale Sampa, Ronald Fisa, Mercy W. Monde, Choolwe Jacobs

**Affiliations:** 1 Clinton Health Access Initiative, Lusaka, Zambia; 2 Women in Global Health Zambia, Lusaka, Zambia; 3 Ministry of Health, Lusaka, Zambia; 4 School of Public Health, University of Zambia, Lusaka, Zambia; 5 Southern African Institute for Collaborative Research and Innovation Organisation, Lusaka, Zambia; 6 Medical Library, University of Zambia, Lusaka, Zambia

**Keywords:** antenatal care, early initiation of ANC, first trimester, interventions, strategies

## Abstract

**Background:**

Despite the availability of antenatal care (ANC) services, fewer than 40% of pregnant women in sub-Saharan Africa initiate ANC within the first trimester, indicating suboptimal early utilisation of maternal health services in the region.

**Aim:**

This review aimed to identify and synthesise evidence on interventions that improve the timely initiation of ANC among pregnant adolescents and women in sub-Saharan Africa.

**Setting:**

All studies included in the review were conducted in sub-Saharan Africa.

**Method:**

A systematic search guided by Preferred Reporting Items for Systematic Review and Meta-Analyses across seven databases (PubMed, Embase, Cumulative Index to Nursing and Allied Health Literature [CINAHL], Scopus, and the Cochrane Database of Systematic Reviews - and grey literature sources including ProQuest Dissertations & Theses Global and African Journals Online [AJOL]) identified eligible English-language studies (1990–2022), including randomised and non-randomised trials, controlled before-and-after studies, and interrupted time-series. The review was conducted between September 2022 and October 2022, and the protocol was registered with PROSPERO (CRD42022380283).

**Results:**

Eleven studies met the inclusion criteria. Three types of intervention approaches were identified: community-based, facility-based, and a combination of both. Facility-based interventions, such as mobile phone reminders, for instance, the wired mothers innovation and quality improvement initiatives, showed improvements in early ANC attendance ranging from a 5% increase to more than triple the uptake. Community-based interventions also proved effective, with gains of up to 200%, as in Malawi, where early ANC increased from 10% to 29%. These mechanisms included home visits, education, male involvement, and engagement with traditional leaders.

**Conclusion:**

Integrated community and facility-based interventions offer promising strategies to improve timely ANC. Future efforts should consider cost-effectiveness and implementation research to enhance decision-making.

**Contribution:**

This study demonstrates the potential of underutilised interventions to enhance first-trimester ANC attendance and maternal health outcomes across sub-Saharan Africa.

## Background

Timely initiation of antenatal care is defined as the first antenatal care (ANC) contact occurring within the first trimester of pregnancy.^1^ Timely initiation of ANC is crucial as it enables pregnant women to benefit from health promotion, disease prevention, and early screening of risk factors and conditions, ensuring optimal health outcomes for both mothers and children.^2,3^

Attendance at ANC during the first trimester allows for the early detection and management of pregnancy-related complications, establishment of baseline clinical information, and provision of counselling on pregnancy danger signs and healthy behaviours. In contrast, delayed initiation of ANC has been associated with missed opportunities for early intervention, increased risk of undiagnosed complications, and delayed referral for emergency obstetric care.^4^

In sub-Saharan Africa, only 38% (95% confidence interval [CI]: 37.8–38.2) of pregnant women attend ANC in the first trimester, with rates ranging from 14.5% in Mozambique to 68.6% in Liberia.^5^ Recent Demographic and Health Surveys show that most countries in the region report less than 50% first trimester ANC attendance, with Zambia at just 37%.^6^

Delayed initiation of ANC is not unique to sub-Saharan Africa and is also prevalent in other low- and middle-income settings. Several factors have been consistently associated with late booking, including maternal age, marital status, parity, prior experiences with ANC services, sociocultural and gender norms, unintended pregnancies, limited awareness of early pregnancy signs, inadequate knowledge of the benefits of early ANC, and perceptions of poor quality of care at health facilities.^7,8,9,10^

Improving the timing of ANC initiation is therefore essential for enhancing maternal and neonatal health outcomes. While numerous interventions have been implemented to address barriers to early ANC attendance, evidence synthesising the effectiveness of these strategies – particularly within the sub-Saharan African context – remains limited. Identifying context-specific interventions that have demonstrated effectiveness is critical to informing policy decisions and guiding governments, global health organisations, and implementing partners in prioritising strategies aimed at reducing maternal morbidity and mortality.

### Aim

This systematic review aimed to identify and synthesise evidence on interventions that improved timely initiation (first trimester) of antenatal care among pregnant adolescents and women in sub-Saharan Africa, as well as factors associated with early ANC initiation in the region.

## Methods

This systematic review was undertaken between September 2022 and October 2022 and was conducted in accordance with the Preferred Reporting Items for Systematic Review and Meta-Analyses (PRISMA) 2020 guidelines^11^ (Appendix 1). The review protocol was registered prospectively with the International Prospective Register of Systematic Reviews (PROSPERO) under registration number CRD42022380283, prior to the commencement of data extraction.

### Criteria for considering studies for inclusion

#### Types of study designs

This review included studies that evaluated interventions aimed at improving timely initiation of ANC – defined as attendance within the first trimester – among pregnant adolescents and women in sub-Saharan Africa. Eligible study designs comprised randomised controlled trials, non-randomised studies, controlled before-and-after (CBA) studies, and interrupted time-series studies, in line with established methodological guidance for systematic reviews of interventions.^1,12^ Only studies published in English between 1990 and 2022 were considered for inclusion.

#### Study participants

Eligibility parameters were defined using the Population, Intervention, Comparison, Outcome (PICOS) framework and presented in the logic model. The review included studies from sub-Saharan Africa focusing on interventions to improve timely ANC initiation (first trimester) among pregnant women, including adolescents and young women (15–49 years). Studies addressing multiple health behaviours were eligible, with no restrictions on baseline maternal health conditions. General population, children, and elderly studies were excluded.

**Types of interventions:** Eligible interventions were those designed to promote or improve initiation of ANC within the first trimester among women of reproductive age in sub-Saharan Africa. Studies were included if they evaluated any intervention that resulted in early ANC attendance or were delivered during the antenatal period with the intention of influencing early care-seeking behaviour, including interventions addressing multiple health behaviours during pregnancy.

**Types of comparators:** Studies that compared multiple health behaviour interventions with non-intervention control, standard of care, or another active intervention addressing one health behaviour were included.

**Types of outcomes:** The primary outcome of interest was early initiation of antenatal care, defined as ANC attendance within the first trimester, as a modifiable health behaviour among pregnant adolescents and women. Outcome data collected using any method – including self-report, direct observation, or objective measures – were considered eligible, including data obtained from service audits and medical or pregnancy records.

### Information sources and search strategy

A comprehensive systematic search was conducted across five electronic databases – PubMed, Embase, Cumulative Index to Nursing and Allied Health Literature (CINAHL), Scopus, and the Cochrane Database of Systematic Reviews – as well as grey literature sources, including ProQuest Dissertations & Theses Global and African Journals Online (AJOL). The final search was completed on 04 October 2022.

The search strategy was developed in consultation with a medical librarian to enhance precision and sensitivity. Search terms included combinations of keywords and controlled vocabulary related to *pregnant women, pregnant adolescents, antenatal care, timely initiation, community-based interventions*, and *sub-Saharan Africa*, using Boolean operators, truncation, and Medical Subject Headings (MeSH) where applicable. Because of language constraints within the research team, only English-language peer-reviewed studies were included. Full search strategies for all databases are included in the Appendix 1.

#### Study selection

Titles and abstracts retrieved through the search were independently screened for eligibility by two reviewers, with disagreements resolved through consultation with a third reviewer (Ronald Fisa, Mutale Sampa and Mercy W. Monde) using the eligibility criteria as prescribed in the study protocol. After screening of titles and abstracts, full texts of potentially eligible studies were retrieved. Two authors (Ronald Fisa and Mutale Sampa) independently screened full texts for eligibility. Study selection and screening were managed in Covidence, a software for managing and streamlining systematic reviews (Covidence systematic review software, 2019). Studies that did not met the criteria were excluded. Disagreements were resolved through discussion with a third reviewer (Choolwe Jacobs). A final decision on the included studies was made, leading to the extraction phase. A PRISMA flow diagram was used to illustrate the screening procedure.

### Data extraction process and quality assessment

Data were independently extracted by two reviewers using a pre-piloted data extraction tool developed in Microsoft Excel. More specifically, screening of the selected articles was performed with a view to collecting data for the categories as seen in Table 1. Data on the effect(s) that each intervention had on early initiation of ANC or early utilisation of ANC was extracted from identified and included studies after the full-text screening. The extracted items included the author’s name, year of publication, country, study design, the interventions considered, factors associated with timely initiation (in the first trimester) of antenatal care, and the key findings of studies.

**TABLE 1:** A summary of extracted studies.

Authors	Country	Purpose	Approach	Intervention	Outcome	Associated Factors and comments
Geldsetzer et al.^14^	Tanzania	To determine the impact of a community health worker (CHW) intervention on the proportion of women who (1) visit ANC fewer than 4 times during their pregnancy and (2) deliver at home	Community-based intervention	Home visits to identify pregnant women and refer them to ANC, counselling pregnant women on maternal health Home visits to women who missed an ANC or PMTCT.	Attending ANC in the first trimester did not differ significantly between study arms (15% in standard care vs 16.1% in intervention arm) at endpoints.	Quantity of CHW conducting visits was insufficient Poor quality of ANC disrespectful and empathetic communication by the providers Long waiting hours Inconveniences or economic burden of attending ANC due to CHW referrals
Ensor et al.^15^	Zambia	To determine whether a complex community intervention in rural Zambia improved understanding of maternal health and increased use of maternal healthcare services	Community-based intervention	Mobilising communities to improve maternal health: improving the effectiveness of Safe Motherhood Action Groups by training volunteers and developing and strengthening systems in the community that help women get to healthcare facilities.	The intervention was associated with significant increases in maternal health indicators, including early ANC (41.4% at baseline and 70% at endline).	Women’s knowledge of ANC and obstetric danger signs, use of emergency transport and deliveries by skilled providers
Hembling et al.^24^	Ghana	To examine the impact of mobilising faith-based and lay leaders to address the sociocultural barriers to antenatal care uptake in northern Ghana in the context of a broader child survival project	Community-based intervention	The CoC intervention; included the formation of village-level councils.	Early ANC and increased (57% baseline and 60% endline) although not statistically significant *p* = 0.05).	Social norms may be barriers High level of community engagement, dialogue and coordination
Kachimanga et al.^16^	Malawi	To evaluate changes in uptake after deployment of CHWs between March 2014 and June 2016	Community-based intervention	The CHWs were switched from a patient-based approach in which CHWs were assigned to a specific group of patients, to a household-based approach in which CHWs were assigned to cover a given number of households.	The proportion of pregnant women starting ANC in the first trimester increased (from 10% to 29% – 200%: 95% CI: 163% – 234%).	Improvement associated with improved reporting and enhanced quality of care at the health facilities
Kawooya et al.^17^	Uganda	To study evaluated change in maternal attendance in the first and fourth ANC visits, the number of births at local healthcare facilities, the number of referrals from lower to higher level healthcare facilities because of ultrasound (US)-aided detection of high-risk complications	Health facility-based intervention	Ultrasound training support and donated US machines.	There was a 32% increase in the first ANC attendance at the intervention sites compared with 7.4% in the control sites.	Integration of diagnostic services such as ultra sound has significant effect on early attendance of ANC
Lema et al.^18^	Tanzania	To improve ANC and PMTCT uptake and retention within the Dares Salaam public-sector health system	Combined community-based intervention and health facility-based intervention	Community Health Workers (CHWs); identifying pregnant women, educating women, routinely visit them and link them to MCH care.	More than 75% of pregnant women were identified to attend ANC in first trimester. ANC and PMTCT uptake increased.	Integration of services, MNH, child health and PMTCT proved useful
Lund et al.^19^	Tanzania	To evaluate the association between a mobile phone intervention and antenatal care in a resource-limited setting aimed to assess antenatal care in a comprehensive way taking into consideration utilisation of antenatal care as well as content and timing of interventions during pregnancy	Facility-based intervention	The ‘Wired Mothers’ Intervention; consisted of an automated short messaging service (SMS) system providing wired mothers with unidirectional text messaging and a mobile phone voucher system providing the possibility of direct two-way communication between wired mothers and their healthcare providers.	The mobile phone intervention was associated with an increase in antenatal care attendance (OR: 2.39; 95% CI: 1.03–5.55).	-
Mwaniki et al.^20^	Kenya	To examine the application of quality improvement to increase utilisation of integrated health services (ANC, PMTCT, and skilled delivery) and improve adherence to clinical standards and guidelines in an entire rural district	Facility-based intervention	Healthcare Improvement (HCI) Project provided 1 week training on quality improvement to the DHMT.	The number of pregnant mothers starting ANC within the first trimester increased from 8% to 24%, *p* = 0/002.	-
Singh et al.^21^	Ghana	To determine whether ‘Project Fives Alive!’ influenced maternal and child health outcomes at scale. A secondary objective is to present a methodology of using facility-based routine health data for a large-scale impact evaluation.	Facility-based intervention	Quality improvement project.	Several MNH categories (early ANC, four or more ANC visits and skilled delivery/PNC) were significantly associated with improved outcomes.	-
Ssetaala et al.^22^	Uganda	To explore whether community health worker household-based maternal health visits improve antenatal care and skilled birth attendance among hard-to-reach fishing villages on Lake Victoria, Uganda	Community-based intervention	Participants from intervention communities received the CHW home visit package.	CHW intervention was associated with attendance of first antenatal care visit within 20 weeks of pregnancy (OR: 2.1 [95% CI: 1.6–7.6].	Level of education was associated with early initiation of ANC CHWs have crucial role in improving ANC, early community-based diagnosis such as anaemia and hypertension
Wafula et al.^23^	Uganda	To assess the effect of community health worker (CHW) involvement on utilisation of antenatal services during pregnancy in resource-constrained rural settings in Uganda	Community-based intervention	Community dialogues and empowering CHWs to educate pregnant women about using maternal health services.	Intervention did not significantly improve early imitation of ANC (DiD = 1.3%).	ANC attendance was associated with posy primary education and high wealth quintile

Note: Please see the full reference list of the article Akinola O, Zimba R, Banda K, et al. A systematic review of implementation strategies to improve timely initiation of antenatal care among pregnant women in sub-Saharan Africa. J Public Health Africa. 2026;17(1), a1461. https://doi.org/10.4102/jphia.v17i1.1461, for more information.

ANC, antenatal care; PMTCT, prevention of mother-to-child transmission; MCH, maternal child health; CoC, Council of Champions; CI, confidence interval; MNH: maternal newborn health; DHMT, District Health Management Team; OR, odds ratio; PNC: postnatal care;

PNC, postnatal care; DiD, difference in difference; QI, quality improvement.

### Risk of bias assessment

Risk of bias was assessed using the Cochrane Risk of Bias 2 (RoB 2) tool for randomised trials and the Risk of Bias in Non-randomised Studies of Interventions (ROBINS-I) tool for non-randomised studies.^11^ Guided by the revised 2019 Cochrane handbook, two authors independently assessed each study, resolving disagreements by consensus. Risk of bias was rated as low, high, or unclear across various domains. For randomised controlled trials (RCTs), domains included bias from the randomisation process, participant recruitment timing, deviations from intended interventions, missing outcome data, outcome measurement, and selective reporting. For non-randomised studies, additional domains such as confounding, participant selection, intervention classification, and sequence generation were evaluated. This comprehensive approach ensured consistent and systematic assessment of implementation and reporting quality across study types.

### Data analysis and synthesis

The first objective aimed to identify and synthesise evidence on interventions that improved timely initiation (first trimester) of antenatal care among pregnant adolescents and women in sub-Saharan Africa. To address this, information was systematically retrieved and collated using numerical, graphical, tabular, and narrative summaries. A descriptive numerical and tabular summary of the total number of included studies was carried out to describe key characteristics of the studies, such as country, year of publication, study design, sample size, and the risk of bias assessment outcomes. Data from randomised and non-randomised study designs were synthesised separately. Intervention characteristics that reported outcomes were identified. The second objective, all examined associated factors were collated and presented in a table. Heterogeneity was assessed and explored by differences between study designs and quality dimensions (RCTs and non-RCTs) while statistical heterogeneity assessed the variation of effects between studies. We found substantial heterogeneity in the identified studies, and meta-analysis was not deemed appropriate.^13^

## Review findings

**FIGURE 1:**
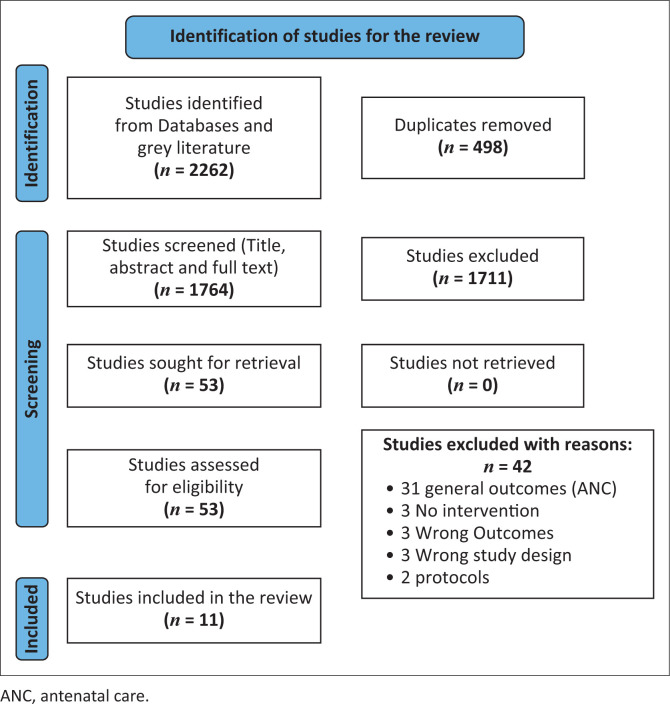
Preferred reporting items for systematic review and meta-analyses flowchart.

A total of 2262 records were identified through the database search (Figure 1). After removal of 498 duplicate records, 1764 studies remained for title and abstract screening. Following this stage, 1711 articles were excluded because they did not focus on first trimester ANC initiation, were study protocols, employed inappropriate study designs, assessed irrelevant interventions, or were not published in English. As a result, 10 studies met the inclusion criteria and were included in the review (Table 1).^14,15,16,17,18,19,20,21,22^ An additional six studies were identified through reference list screening, of which one study was eligible for inclusion.^23^ The study selection process is illustrated in the PRISMA flow diagram (Figure 1).

### Characteristics of included studies

Four of the studies were published in 2014.^15,18,19,20^ and the rest were published between 2015 and 2022.^16,17,21,22,23,24^ Three studies were conducted in Tanzania,^14,18,19^ three in Uganda,^17,22,23^ two in Ghana^21,24^ and the rest with one in each country. None of the studies were multicountry studies.

Regarding the type of studies, two studies were cluster randomised trials,^14,18^ eight were quasi-experimental studies^15,16,17,19,20,21,23,24^ and one was a pragmatic comparative study.^17^

Among the eleven studies reviewed, six used community-based interventions (CBIs),^14,15,16,22,23,24^ three were facility-based,^17,20,21^ and two combined both approaches.^18,19^ Sample sizes for women ranged from 1970^22^ to 3405^14^; for healthcare providers or community nurses, from 31^14^ to 744^21^; and for community health workers (CHWs), from 211^15^ to 215.^14^ Some studies did not report provider sample sizes. All interventions (Table 1) aimed to improve first trimester ANC attendance as part of broader maternal newborn health (MNH) efforts.

### Approaches of intervention

#### Community-based interventions

Community-based interventions were delivered through diverse mechanisms, including community outreach,^15,23,24^ home visits,^14,16,18,22,24^ community dialogues^14,23,24^ and through referral of mothers to the health facilities.^15,18^

The distribution points ranged from homes^14,15,16,19,22,23,24^ to community centres,^15,16^ village centres,^24^ waiting homes^16^ to health facilities.^14^ The most popular, and perhaps relatively systematic, approach to distributing care was home visits. However, home visits were complemented with escorts to health facilities.

**Mechanisms for community-based interventions:** Community health volunteers and outreach nurses were trained to identify pregnant women through home visits, offering education and counselling on preventive care and maternal health services, including prevention of mother-to-child transmission (PMTCT).^14,15,22,23^ Both existing and newly trained CHWs supported women to attend health facilities. Household-based approaches, where CHWs were assigned specific households or participants, proved more effective in Malawi^16^ and Uganda.^22^

Notably, CHWs had a responsibility of connecting identified women to the health facilities by either escorting them to the facilities^14^ or encouraging them to do so.^15^ While homes may be an important location for the provision of services, the linkage to health facilities is crucial in dealing with referral and management of those who need more professional care.^25^

Some studies reported integration of other health services, such as PMTCT programmes^14,16,18^ and a broader child survival programme.^24^ For some studies, training, education, and awareness is presented as part of many other interventions, while for other studies these components are used to optimise other interventions. Several components and pieces are added to the home visits mechanism, depending on the type and purpose of the intervention. The CHWs visited women enrolled in PMTCT care and provided information and counselling.

In Tanzania^14^ CHWs visited women enrolled in PMTCT care and provided information and counselling. In their study, Geldsetzer et al.^14^ used trained CHWs to revisit pregnant women at home to verify whether they had attended ANC and to explore who missed an ANC or PMTCT and understand why and to encourage them to attend.

The study by Lema et al.^18^ is also another example of continuous support for women, in which routine revisits in all identified pregnant women were conducted within 2 weeks after the initial visit to verify whether women had visited ANC. Lema et al.^18^ argues that through revisits, CHWs became increasingly effective in identifying pregnant women in the community. This observation could be explained by the fact that CHWs might have learned how to approach women and families in the community best and elicit information on pregnancy status.

**Provision of tools for implementing interventions:** Several reviewed studies highlighted the importance of providing additional support to implementers to improve ANC outcomes. This support included equipment, supplies, transport, and infrastructure.^14,15,19,22^ In Tanzania, registers were given to CHWs for tracking household visits to pregnant women.^14^ In Zambia, community transport suited to local terrain was provided,^15^ while in another study, bicycle ambulances eased access to health facilities during obstetric emergencies.^24^ In Zanzibar, mobile phones were used to send short messaging service (SMS) reminders for ANC, which increased first trimester attendance.^19^

In Uganda, CHWs received essential supplies, supervision, and refresher training to support early diagnosis and management of hypertensive disorders in pregnancy.^22^ This intervention was found to be cost-effective across the settings.^26^ In Malawi, maternity waiting homes were constructed and an additional nurse was hired to meet rising demand at intervention facilities.^16^ This led to a 200% increase in the proportion of women initiating ANC early. These interventions suggest that targeted logistical and material support to implementers – both at community and facility levels – can significantly improve ANC uptake and service quality, especially when adapted to local needs and supported by health system strengthening measures.

Additional support for care by implementers of interventions is another mechanism being used in some reviewed studies. The support to implementers came in the form of equipment, supplies and materials.^14,15,19,22^ In Tanzania, Geldsetzer et al.,^14^ provided registers to CHWs for monitoring CHWs’ activities in the intervention wards by logging in each household visit to a pregnant woman. In Zambia, Ensor and colleagues’s^15^ intervention was to provide community transport to groups of two or three villages. While in a study by Hembling et al.,^24^ bicycle ambulances were also provided to ease transport from the community to the facility during obstetric emergencies.

#### Facility-based interventions

Facility-based interventions form the cornerstone of current health systems. With the growing demand for quality facility-based services, evidence of facility-level interventions to improve initiation of ANC within the first trimester was explored in articles under review. We found two quality improvement studies, one in Ghana^21^ and another in Kenya^20^ that implemented interventions aimed at improving the timeliness, completeness and accuracy of the data being submitted to and reported by the District Health Information Management System (DHIMS) offices, in efforts to improve maternal and child health outcomes at a scale.

Another facility-based intervention conducted in Tanzania^19^ was aimed to evaluate the association between a mobile phone intervention named ‘wired mothers’ and ANC in Zanzibar and hypothesised that the ‘Wired Mothers’ intervention can increase ANC attendance as well as improve content and timing of antenatal care services provided to individual women.

**Mechanisms for facility-based interventions:** Two of the articles described interventions that employed an approach of quality improvement^20,21^ trained District Health Management Team (DHMT) officers as implementers. Both studies were non-randomised studies that routinely collected maternal newborn, child health (MNCH) data in the facilities and involved training of key personnel in quality management of data.

Mwaniki et al.^20^ implemented a quality improvement intervention to increase utilisation of integrated health services (ANC, PMTCT, and skilled delivery) in an entire rural district that was performing below the national average in most of the health indicators over a 20-month period. Trained DHMT members were assigned to two or three 7–12-member quality improvement teams composed of facility healthcare personnel, community healthcare volunteers, and community representatives to mentor/coach them on quality improvement as part of their regular supportive supervision. Primary indicators in the intervention were ANC.

Similarly, Singh et al.,^21^ in their quality improvement intervention in Ghana, aimed at improving the health outcomes of mothers, infants, and children under-5 years by improving the coverage, quality, reliability and patient-centredness across all public and faith-based facilities in Ghana. In this vein, a quality improvement team in each facility was formed, and each member attended four learning sessions and shared progress with other quality improvement (QI) teams. In addition, supervision and coaching visits were made by project staff in conjunction with district health supervisors.

This approach entailed the identification of process failures by health staff and testing these process changes in the health facilities to address those failures^27^ seemed to have been effective as demonstrated in Sigh et al.,’s study^21^ where there were some improvements in maternal health outcomes over time, with an increase in early ANC from 37% to 42%.^21^

In Tanzania, Nielsen et al.,^19^ implemented mobile phone innovative training initiatives known as the Wired Mothers’ Intervention, consisting of an automated short messaging service (SMS) system providing wired mothers with unidirectional text messaging between wired mothers and their primary healthcare providers. The aim of the SMS component was to provide simple health education and appointment reminders to encourage attendance at routine ANC, skilled delivery attendance, and postnatal care.

This intervention demonstrated the efficacy of mobile phones in improving ANC and also made remembrance of ANC appointments easier, which can be helpful even in different contexts.^19^ Furthermore, Nielsen et al.^19^ argued that mobile phones are an economic innovation that could influence the success or outcome in similar contexts.

#### Integration and linkage of services, including referral

A combination of care providers and strategies within the community and health facilities has been used in the implementation of interventions. According to Geldsetzer et al.,^14^ the intervention engaged both CHW and healthcare providers, known as community outreach nurses, who were tasked with additional maternal healthcare tasks. Community outreach nurses to whom women were referred by CHW for ANC facilitated and monitored the referral system and verified documentation for referred mothers at healthcare facilities, hence adding to supervision and support to CHWs.^14^

In a study by Hembling et al.^24^ village-level councils comprised of faith-based leaders (Protestant ministers, traditional African religious leaders, and Islamic imams), village chiefs, traditional medical practitioners, and female leaders known as ‘queen mothers’ or ‘*magazia*’. Using this approach of engaging community leaders who are highly influential and respected leaders as custodians of traditional practices, attitudes, and beliefs related to MNCH was assumed as the best approach in addressing deeply held norms and traditional practices and promoting behavioural changes throughout the community.^24^

Tudor^28^ argues that integration of services can reduce programme costs and time through more synergistic use of resources. Integrating ANC services with other services can be realised with involvement of all stakeholders and consideration of the health system and socio-political contexts.^29^ However, if well thought out integration can have undesirable consequences, particularly in low-resourced contexts with already constrained and overloaded health systems.^30^ Therefore, integrating and linking services need to be carefully considered as they introduce complexity (and uncertainty) to studies and related outcomes.

#### Outcomes or effects of community and facility-based interventions

Table 2 provides a summary of the interventions and outcomes from the reviewed articles. All the interventions demonstrated a significant increase in attendance of ANC in the first trimester, except for two; by Wafula et al.,^23^ in Ghana (Difference in Difference = –1.3%) and by Geldsetzer et al.,^14^ (59.1% versus 60.7% between intervention and standard-of-care care arm; risk ratio [RR] 0.97; 95% CI: 0.82–1.15; *p* = 0.754) in Tanzania. The rest of the articles demonstrated a significant increase in attendance of ANC in the first trimester.

**TABLE 2:** Risk of bias assessment in randomised controlled studies.

Serial number	Author	Bias arising from the randomisation process	Bias arising from the timing of identification or recruitment of participants	Bias because of deviations from intended interventions	Bias because of missing outcome data	Bias in measurement of the outcome	Bias in selection of the reported result	Overall bias
1	Geldsetzer et al.^12^	Low risk	Low risk	Low risk	Low risk	Serious risk	Low risk	Serious risk
2	Lema et al.^32^	Low risk	Serious risk	Low risk	Low risk	Serious risk	Low risk	Serious risk
3	Lund et al.^9^	Some concern	Low risk	Low risk	Low risk	Serious risk	Low risk	Serious risk

Note: Please see the full reference list of the article Akinola O, Zimba R, Banda K, et al. A systematic review of implementation strategies to improve timely initiation of antenatal care among pregnant women in sub-Saharan Africa. J Public Health Africa. 2026;17(1), a1461. https://doi.org/10.4102/jphia.v17i1.1461, for more information.

All the facility-based interventions improved the early initiation of ANC. For instance, a significant increase in early ANC attendance (odds ratio [OR]: 2.39; 95% CI: 1.03–5.55) was observed in a facility-based intervention called the wired mothers, using mobile phones to send reminders,^20^ while in a quality improvement intervention by Mwaniki et al.,^20^ in Kenya, a triple effect (8% to 24% *p* = 0.002) was observed between the baseline and end line, and another quality improvement intervention by Singh et al.,^20^ revealed an increase in early ANC from 37% at pre-intervention to 42% at post intervention.

Notably, community-based interventions that were behavioural related increased attendance of ANC in the first trimester, except for a study by Wafula et al.,^23^ and Geldsetzer et al.,^14^ For instance, a Malawi study,^17^ using a household approach integrating other services such as TB and HIV in addition to maternal health services had a significant triple effect (from 10% to 29% – 200%: 95% CI: 163% – 234%) on improving ANC attendance within the first trimester. This intervention managed to triple the proportion of women starting ANC in the first trimester. Most of these interventions also increased the proportion of women completing at least four ANC visits as well as attending skilled birth attendance.

### Risk of bias assessment

Of the eleven articles included in this review, eight articles were quasi-experimental designs and three were purely cluster-randomised studies. Because of the nature of different study designs, we used two quality assessment tools, Cochrane Risk of Bias assessment 2 (ROB2) and Risk of Bias in Non-randomised Studies (ROBINS-I) of Interventions.

#### Cochrane risk of bias assessment

Using the ROB2, all three RCT studies^14,18,19^ were assessed and their risk of bias was found to be of high risk. Some of the observations made from these studies were that these RCTs did not blind the participants as well as health workers because of the nature of the interventions. In these studies, the allocation sequence was not concealed from either the health workers or the participants until clusters were enrolled and assigned to interventions. Furthermore, one study^19^ had problems in the randomisation of participants to the intervention and control. In this article, it was not clear whether the allocation sequence was concealed until clusters were enrolled and assigned to interventions.

The articles, therefore, scored poorly in these domains of the ROB assessment tool, affecting the overall ROB result. All three articles^14,18,19^ had a high risk of bias in the measurement of the outcome.

A summary of the risk assessment for all the domains is given in Table 2.

#### The risk of bias in non-randomised studies of interventions

In assessing the quality of studies, we used the Risk of Bias in Non-randomised Studies (ROBINS-I) of Interventions. For the overall risk of bias for these studies, three of the articles^20,21,24^ were found to have low risk bias and five^15,16,17,22,23^ were found to be with moderate risk of bias. Table 3 gives a summary of the risk of bias assessments for all the 8 studies in all the domains.

**TABLE 3:** Risk of bias assessment for non-randomised studies for the different domains.

Author	Bias because of confounding	Bias in selection of participants into the study	Bias in classification of interventions	Bias because ofdeviations from intended interventions	Bias because ofmissing data	Bias in measurement of outcomes	Bias in selection of the reported result	Overall risk
Ensor et al.^15^	Moderate risk	Low risk	Low risk	Low risk	Low risk	Moderate risk	Low risk	Moderate risk
Hembling et al.^24^	Low Risk	Low risk	Low risk	Low risk	Low risk	Moderate risk	Low risk	Low risk
Kachimanga et al.^16^	Moderate risk	Low risk	Low risk	Low risk	Critical risk	Moderate risk	Low risk	Moderate risk
Kawooya et al.^17^	Moderate risk	Moderate risk	Low risk	Low risk	Serious risk	Moderate risk	Moderate risk	Moderate risk
Mwaniki et al.^20^	serious risk	Low risk	Low risk	Low risk	Low risk	Low risk	Low risk	Low risk
Singh et al.^21^	Low risk	Low risk	Low risk	Low risk	Low risk	Low risk	Low risk	Low risk
Ssetaala et al.^22^	Low risk	Low risk	Moderate risk	Low risk	Moderate risk	Moderate risk	Low risk	Moderate risk
Wafula et al.^23^	Moderate risk	Low risk	Moderate risk	Low risk	Low risk	Moderate risk	Low risk	Moderate risk

Note: Please see the full reference list of the article Akinola O, Zimba R, Banda K, et al. A systematic review of implementation strategies to improve timely initiation of antenatal care among pregnant women in sub-Saharan Africa. J Public Health Africa. 2026;17(1), a1461. https://doi.org/10.4102/jphia.v17i1.1461, for more information.

#### Risk of bias assessment by domain in non-randomised intervention studies

**FIGURE 2:**
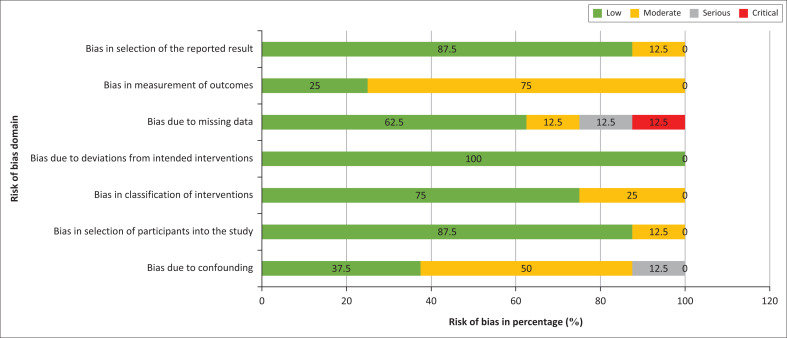
Risk of Bias in Non-randomised Studies of Interventions risk of bias assessment for intervention studies.

A summary of the risk of bias is given in Figure 2. Overall, almost all the articles had low risk of bias. All the studies in the review did not deviate from the intended interventions of the study. Only one study^17^ representing 12.5% of the studies had bias in the selection of participants, the reported result, and a serious risk of bias because of confounding,^20^ moderate risk of bias in the selection of participants into the study^17^ and a serious risk of bias because of missing data,^17^ only a small fraction of the articles had a critical risk of bias in missing data.^16^

The majority (87.5%) of the studies^15,16,20,21,22,23,24^ had a low risk of bias in the selection of participants. Low risk of bias in measurement was observed in only 25% of the studies.^20,21^

Notably, 75% of the studies had a low risk of bias in the classification of the intervention^15,16,20,21,24^ while 87.5% had a low risk of bias in the selection of participants^15,16,20,21,24^ About 12.5%^20^ of the studies with a serious risk of bias.

Bias because of missing data was observed in 25% of the studies. Overall, majority of articles had low risk of bias for most of the articles.

## Discussion

This systematic review synthesised evidence from 11 studies that evaluated interventions aimed at improving timely initiation of ANC in sub-Saharan Africa. The majority of the included studies demonstrated positive effects on early ANC attendance, with many interventions incorporating training and support for health providers. Community-based approaches – most commonly implemented through trained and supervised CHWs conducting home visits – were particularly influential in increasing awareness and encouraging women to initiate ANC early.^31,32^

Interventions that supported implementers with equipment, supplies, and materials, particularly in Tanzania, Malawi, and Uganda, also showed strong effects on early ANC uptake.^14,15,19^ Evidence suggests that bundled interventions addressing multiple maternal and newborn health issues are more effective than single interventions.^33^ The review also highlights the importance of community engagement and local leadership support for successful implementation. Acceptability of community-based approaches by local stakeholders, including village chiefs and female leaders was crucial.^24^

Studies underscore the need for supportive systems such as community-arranged transport to sustain CHW engagement and service delivery.^34^ Behavioural interventions targeting marginalised populations are inherently complex and require context-specific tools and strong community support structures.^35^ Overall, the findings of this study indicate that integrated, community-driven interventions backed by adequate resources and stakeholder involvement can significantly improve early ANC initiation and contribute to better maternal health outcomes in low-resource settings.^3^

The use of mobile phones in healthcare has become an increasingly prioritised strategy for strengthening health systems. The positive impact of behavioural interventions to increase early attendance adherence in this review using phones observed in this review is in line with previous work documenting the effects of reminders using mobile health interventions on ANC adherence and maternal and neonatal health outcomes^25,36,37^ Lund et al.’s^19^ phone-based interventions known as wired mothers aimed at sending automated texts to mothers to create awareness and reminders for ANC found the effect of their interventions because the interventions targeted mothers’ high motivation to adhere to ANC/postnatal care (PNC) regimens, as well as cognitive processes vital to adherence, such as memory monitoring.^37^ Mobile phone applications may be considered particularly in resource-limited settings.

Notable improvements in early initiation of ANC among pregnant women were observed through the implementation of quality improvement initiatives.^20,21^ These approaches focused on building the capacity of healthcare providers through training and ongoing coaching in the use of routinely collected health data to enhance data completeness, timeliness, and accuracy within the District Health Information Management System (DHIMS). Strengthening data-driven decision-making in this way contributed to improved leadership capacity among providers and enabled more effective utilisation of limited health system resources, leading to better service delivery outcomes.

The intervention implemented in Kwale District^20^ emphasised the integration of ANC and PMTCT services, while actively engaging communities through structured dialogue platforms. These community dialogues created opportunities for stakeholders to provide feedback on critical service delivery issues, including reports of mistreatment during childbirth, and supported the identification and implementation of corrective actions at both health facility and catchment-area levels.

This approach created a unique continuum of care in a rural district as facilities together with the community would develop, plan and test any agreed changes. According to Kananura et al.^38^ community dialogues enhance the ability of stakeholders to hold each other accountable. Other researchers argue that community dialogues strengthen appropriate decision-making, advocacy and resource sharing.^39,40^ On the other hand, while interventions that integrated services in Kenya and Ghana demonstrated effective means to improve access to ANC services, if not well taken care of, integrated services can lead to a burden on healthcare providers, particularly in rural facilities with limited resources.

In this systematic review, we have established that factors influencing low uptake of early ANC include education level, maternal employment status, household economic status, distance to health facility, parity and maternal age.^16,24^ These results are similar to those found by Seidu,^41^ in which he looked at factors associated with early antenatal care attendance among women in Papua New Guinea and found that working women had about 1.37 times higher odds of early ANC attendance compared with those who were not working.

Our review has also found that engagement of different leaders in the community, such as community leaders, faith-based leaders, and men, especially husbands, is helpful in encouraging women to attend ANC early, as they know they are pregnant.^24^ Owusu^42^ in his study in Ghana found that husbands were poorly involved in ANC services in the Sunyani municipality. There is need to sensitise the men so that they are involved more as they will in turn encourage their wives for go for this service.

While most interventions reviewed effectively improved early ANC initiation, future efforts should assess scalability and cost-effectiveness. Robust implementation teams, including health economists, are needed to evaluate sustainability. Engaging women’s groups, men, and community leaders can strengthen empowerment and long-term impact of maternal health interventions across various contexts.

A wide range of interventions have been implemented across sub-Saharan Africa; however, gaps remain in addressing sociocultural barriers such as traditional beliefs and religious norms. Future interventions should tackle broader socioeconomic and structural issues such as education, income, and livelihoods, which are strongly linked to poorer maternal health outcomes.

### Limitations

This review highlights key limitations: no identified interventions specifically targeted adolescents, despite their inclusion in the study aim. Additionally, there was limited evidence on factors influencing early initiation of ANC, indicating a gap in research that must be addressed to design more targeted and effective interventions.

This limitation is expected, as the secondary objective may not have aligned well with the search criteria. In addition, a few studies described complex interventions linking households to health facilities through referral and information systems, with the exception of Mwaniki et al.,^20^ highlighting a gap in the integration of continuum of care approaches.

## Conclusion

Although early ANC initiation faces common challenges, certain health facilities, regions, or countries may achieve better outcomes by implementing distinctive and effective community- or facility-level practices. This systematic review found good evidence that community and facility-based interventions can improve early initiation of ANC in the first trimester. This review found that community interventions that targeted behaviour change interventions improvement by large proportion in ANC initiation in the first trimester. It is important to acknowledge the untapped potential of these strategies in promoting early ANC attendance, which is crucial for improving maternal health outcomes.

### Suggestions for future research

Future interventions should also factor cost-effectiveness into the approaches. Robust implementation teams that include health economists and anthropologists to be able to measure, model and predict major dimensions of high-impact interventions are critical to be able to report on the potential and measures for sustainability.

## Appendix 1

### PRISMA-P Checklist

#### PRISMA-P 2015 Checklist

**File 1:** PRISMA-P (Preferred Reporting Items for Systematic review and Meta-Analysis Protocols) 2015 checklist.

Section/topic	Number	Checklist item	Information reported		Line number(s)
			Yes	No	
**ADMINISTRATIVE INFORMATION**					
**Title**					
Identification	1a	Identify the report as a protocol of a systematic review	-	-	1-2
Update	1b	If the protocol is for an update of a previous systematic review, identify as such	-	-	
Registration	2	If registered, provide the name of the registry (e.g. PROSPERO) and registration number in the Abstract	-	-	38 and 91
**Authors**					
Contact	3a	Provide name, institutional affiliation, and e-mail address of all protocol authors; provide physical mailing address of corresponding author	-	-	1-10
Contributions	3b	Describe contributions of protocol authors and identify the guarantor of the review	-	-	212-215
Amendments	4	If the protocol represents an amendment of a previously completed or published protocol, identify as such and list changes; otherwise, state plan for documenting important protocol amendments	-	-	Not applicable
**Support**					
Sources	5a	Indicate sources of financial or other support for the review	-	-	217-218
Sponsor	5b	Provide name for the review funder and/or sponsor	-	-	Not applicable
Role of sponsor/funder	5c	Describe roles of funder(s), sponsor(s), and/or institution(s), if any, in developing the protocol	-	-	Not applicable
**INTRODUCTION**					
Rationale	6	Describe the rationale for the review in the context of what is already known	-	-	83-84
Objectives	7	Provide an explicit statement of the question(s) the review will address with reference to participants, interventions, comparators, and outcomes (PICO)	-	-	97-98
**METHODS**					
Eligibility criteria	8	Specify the study characteristics (e.g. PICO, study design, setting, time frame) and report characteristics (e.g. years considered, language, publication status) to be used as criteria for eligibility for the review	-	-	119-127
Information sources	9	Describe all intended information sources (e.g. electronic databases, contact with study authors, trial registers, or other grey literature sources) with planned dates of coverage	-	-	103-112
Search strategy	10	Present draft of search strategy to be used for at least one electronic database, including planned limits, such that it could be repeated	-	-	233
**STUDY RECORDS**					
Data management	11a	Describe the mechanism(s) that will be used to manage records and data throughout the review	-	-	-
Selection process	11b	State the process that will be used for selecting studies (e.g. two independent reviewers) through each phase of the review (i.e., screening, eligibility, and inclusion in meta-analysis)	-	-	-
Data collection process	11c	Describe planned method of extracting data from reports (e.g. piloting forms, done independently, in duplicate), any processes for obtaining and confirming data from investigators	-	-	-
Data items	12	List and define all variables for which data will be sought (e.g. PICO items, funding sources), any pre-planned data assumptions and simplifications	-	-	-
Outcomes and prioritisation	13	List and define all outcomes for which data will be sought, including prioritisation of main and additional outcomes, with rationale	-	-	-
Risk of bias in individual studies	14	Describe anticipated methods for assessing risk of bias of individual studies, including whether this will be performed at the outcome or study level, or both; state how this information will be used in data synthesis	-	-	-
Synthesis	15a	Describe criteria under which study data will be quantitatively synthesised	-	-	-
	15b	If data are appropriate for quantitative synthesis, describe planned summary measures, methods of handling data, and methods of combining data from studies, including any planned exploration of consistency (e.g. *I*^2^, Kendall’s tau)	-	-	-
	15c	Describe any proposed additional analyses (e.g. sensitivity or subgroup analyses, meta-regression)	-	-	-
	15d	If quantitative synthesis is not appropriate, describe the type of summary planned	-	-	-
Meta-bias(es)	16	Specify any planned assessment of meta-bias(es) (e.g. publication bias across studies, selective reporting within studies)	-	-	-
Confidence in cumulative evidence	17	Describe how the strength of the body of evidence will be assessed (e.g. GRADE)	-	-	-
